# Total Body Fat Content versus BMI in 4-Year-Old Healthy Swedish Children

**DOI:** 10.1155/2013/206715

**Published:** 2013-03-31

**Authors:** Elisabet Forsum, Eva Flinke Carlsson, Hanna Henriksson, Pontus Henriksson, Marie Löf

**Affiliations:** ^1^Department of Clinical and Experimental Medicine, Linköping University, 581 85 Linköping, Sweden; ^2^Department of Biosciences and Nutrition, Karolinska Institute, Novum, 141 83 Huddinge, Sweden

## Abstract

Childhood overweight and obesity, a worldwide problem, is generally identified using BMI (body mass index). However, this application of BMI has been little investigated in children below 5 years of age due to a lack of appropriate methods to assess body composition. Therefore, we used air displacement plethysmography (ADP) to study 4.4-year old boys and girls since this method is accurate in young children if they accept the requirements of the measurement. The purpose was to analyze the relationship between BMI and body fat in these children. Body composition was assessed in 76 (43 boys, 33 girls) of the 84 children brought to the measurement session. Boys and girls contained 25.2 ± 4.7 and 26.8 ± 4.0% body fat, respectively. BMI-based cut-offs for overweight could not effectively identify children with a high body fat content. There was a significant (*P* < 0.001) but weak (*r* = 0.39) correlation between BMI and body fat (%). In conclusion, requirements associated with a successful assessment of body composition by means of ADP were accepted by most 4-year-olds. Furthermore, BMI-based cut-offs for overweight did not effectively identify children with a high body fatness and BMI explained only a small proportion of the variation in body fat (%) in this age group.

## 1. Introduction

Childhood overweight and obesity is a growing problem worldwide which, according to the WHO, represents one of the most serious challenges to human health in this century [1]. Globally as many as 42 million children under the age of five were overweight in 2010 [1]. Thus early childhood obesity-prevention interventions represent a rapidly growing research area [2]. For example, Manios [3] has described how a team of 15 partners across the EU are working to develop such a program for children aged 4–6 years. In the USA, Fitzgibbon et al. [4] conducted a pilot intervention study to prevent obesity in 3–5-year-old Latino children, and Taveras et al. [5] tested an intervention in primary care pediatrics including children aged 2–6 years in an attempt to reduce their overweight and obesity. Identification of overweight and obesity in young children is generally based on the BMI (body mass index) of boys and girls from several countries with age- and sex-specific cut-off values for these conditions [6]. However, obesity is characterized by excessive body fat accumulation, and in adults the body fat content for any particular BMI-value is quite variable [7]. Published data suggest that BMI is an inaccurate estimate of body fatness of individuals also in pediatric populations [8]. However, the relationship between BMI and body fatness has been little studied in children below the age of five and no data are available to demonstrate how well the commonly used definition of overweight identifies children with a high body fat content in this age group. This lack of data is likely due to a lack of appropriate body composition methodology. It is therefore of interest to note that the air displacement plethysmography (ADP) technique, a method known to be able to assess body composition accurately in adults [9], has recently been modified for young children. A validation study [10] demonstrated that this method can be accurate also in such subjects provided that measurements are appropriately conducted, which requires that the child accepts to sit alone in a closed chamber during three measurements each with a duration of about 50 seconds. Unfortunately, this requirement makes it difficult to study children below two years of age. Better compliance can be expected among older children, but it is likely that a certain number of children below the age of five will refuse participation. The aims of this paper were (a) to report the compliance of 4-year-old children when performing the ADP measurement according to established requirements; (b) to describe body fatness, assessed by means of ADP, in a group of healthy 4-year-old boys and girls in relation to commonly used BMI cut-off values for overweight; (c) to assess the relationship between BMI and body fat (%) in 4-year-old boys and girls.

## 2. Subjects and Methods

### 2.1. Subjects

Parents who had participated with their children (*n* = 110) in a previous study [11] were asked to let their children participate in the present study and 84 parent couples accepted. The research ethics committee in Linköping approved the study.

### 2.2. Body Composition

Body volume and density along with body fat were evaluated by means of ADP using the pediatric option with software 5.2.0 (Bod Pod Body Composition System, COSMED USA) [10]. In this procedure body mass is measured using an electronic scale and body volume is assessed in a closed chamber utilizing the relationship between pressure and volume. The principle of the measurement is the same as that for adults [13]. However, volume measurements were always performed in triplicate and strictly according the manufacturer's instructions. Corrections for surface area artifact and thoracic gas volume and calculations of body composition were conducted as described by Fields and Allison [10]. 

### 2.3. Weight Status

BMI (kg/m^2^) of boys and girls was calculated. Overweight was assessed according to the International Obesity Task Force [6] using age- and-sex specific cut-off values.

### 2.4. Statistical Analysis

Linear regression analysis was used. Pearson correlation coefficient was calculated and tested for significance. Our sample size (*n* = 76) was sufficient to identify a correlation between BMI and body fat (%) of 0.28 as significant (*P* < 0.05) with a power of 0.8. The comparison of correlation coefficients was based on Fisher's *z* transformation. Significance (2-sided) was accepted when *P* < 0.05.

## 3. Results

**Figure 1:**
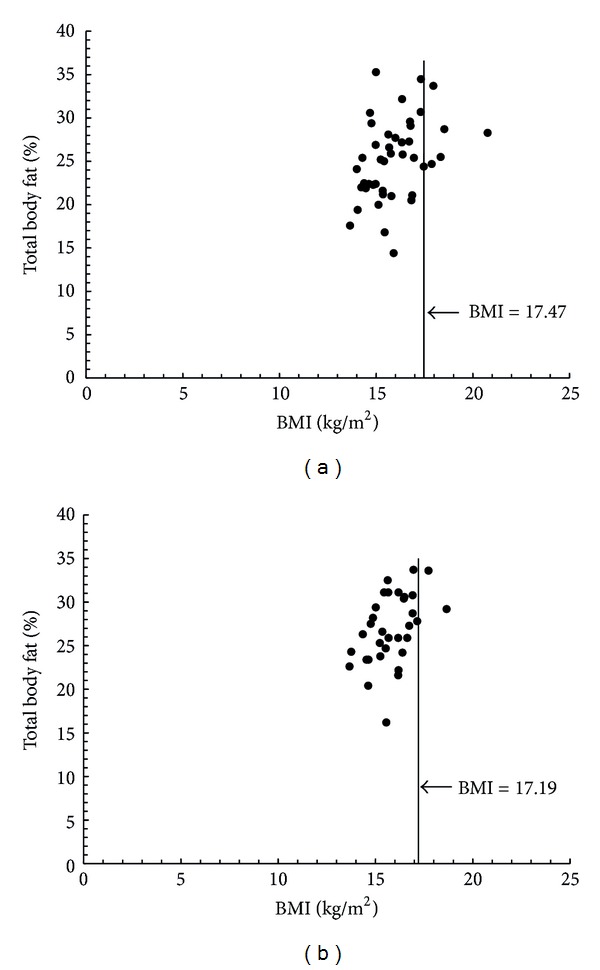
Body mass index (BMI) (kg/m^2^) (*x*) versus total body fat (%) (*y*) in healthy Swedish 4-year-olds in relation to the age- and sex-specific cut-offs for overweight [6], (a) boys (*n* = 43) with overweight cut-off 17.47, (b) girls (*n* = 33) with overweight cut-off 17.19.

Body composition was successfully measured in 76 children, equivalent to 90% of the children brought to the examination. These children are described in Table 1. It should be noted that their weight and height are comparable to Swedish reference data as demonstrated by the *z*-scores given in this table.  Figure 1 shows BMI versus body fat (%) for boys (a) and girls (b) in the study together with the appropriate age- and sex-specific cut-offs for overweight. Obviously, children with a high body fat content may well have a BMI below the cut-off for overweight and children with a BMI above this cut-off may well have a comparatively low body fat content. For example, children with a BMI between 15 and 16 had a body fat content ranging from 14.3 to 32.5%. BMI (*x*) and body fat (%) (*y*) were significantly but weakly correlated in boys (*r* = 0.38, *P* < 0.01, *y* = 1.22*x* + 5.74), in girls (*r* = 0.46, *P* < 0.01, *y* = 1.66*x* + 0.63), and in the sexes combined (*r* = 0.39, *P* < 0.001, *y* = 1.32*x* + 4.94). The correlation coefficients for boys and girls were not significantly different. 

## 4. Discussion

In this study of 4-year-old boys and girls we found that most children accepted the requirements of the ADP measurement and that BMI-based cut-offs for overweight did not effectively identify children with a high body fat content. We also found weak but significant correlations between BMI and body fatness in boys and girls. 

In our study a large proportion of the children brought to the investigation by their parents could be measured with ADP. It is relevant to point out that these parents represented quite a special group since they had previously agreed to participate in a study [11] when their children were newborns. Therefore they represented a selected group that tended to be quite positive towards participation in research. On the other hand, most of them, fathers as well as mothers, were professionally active with busy schedules which may have diminished their possibility to participate with their children in the study. Therefore, our parent population is not necessarily comparable to other parent populations regarding the proportion willing to accept participation in studies. In spite of these considerations, it is important to note that our sample of children is similar to that of healthy Swedish children in general regarding weight and height. In conclusion, our study demonstrated that a large proportion of healthy 4-year-olds who are brought to a measurement session by their parents will accept the requirements of the ADP technique. 

Our study clearly demonstrates that BMI-based cut-offs for overweight do not effectively identify 4-year-old children with a high body fat content. However, a significant correlation between BMI and body fat (%) was found in these children. This relationship appeared to be slightly stronger in girls than in boys but our study may have been too small to identify such a significant difference between the sexes. However, the correlation coefficient for the sexes combined, 0.39, indicates that BMI explained only about 15% of the variation in body fat (%). The corresponding figure for adults is 50–70% [14] and 34–70% for 3–18 year old children [8]. These studies [8, 14] clearly demonstrated that BMI is a poor predictor of the body fat content of individual subjects. The poor correlation between BMI and body fat (%) found in the present study show that this is also the case for 4-year-old children. This finding is likely to have important implications for studies of overweight and obesity in children. For example, our results motivate attempts to include body composition assessment by means of ADP in obesity prevention programs for young children. It is important to realize that such assessments must be properly carried out and that behavioral issues are likely to be a limiting factor in infants and in some young children [15]. Nevertheless, most 4-year-olds accepted the requirements of ADP and therefore this technique can certainly be applied in a large number of young children. Thus it has the potential to be a useful complement to BMI and thereby improve our understanding regarding the biology of childhood obesity development. 

In conclusion, requirements associated with successful assessments of body composition by means of ADP, a valid body composition method, were accepted by most 4-year-old children. Therefore this method has the potential to be a useful complement to BMI in studies related to overweight and obesity in this age group. Furthermore, our study showed that BMI-based cut-offs for overweight do not effectively identify 4-year-old children with a high body fat content and that BMI explains only a small proportion of the variation in body fat (%) in this age group.

## Figures and Tables

**Table 1:** Characteristics of boys and girls studied for body composition by means of air displacement plethysmography (boys = 43, girls = 33).

	Boys	Girls
Age at measurement (yr)	4.42 ± 0.09^1^	4.41 ± 0.03^2^
Weight (kg)	18.9 ± 2.2	18.1 ± 2.2
Weight *z*-score^3^	0.08 ± 1.03	−0.06 ± 0.94
Height (cm)	109 ± 4	107 ± 4
Height *z*-score^3^	0.15 ± 0.81	−0.12 ± 0.98
BMI (kg/m^2^)	15.9 ± 1.5	15.8 ± 1.1
Body fat (%)	25.2 ± 4.7	26.8 ± 4.0

Values are means ± standard deviations. BMI: body mass index.

^1^Range: 4.34–4.96 yr.

^2^Range: 4.34–4.48 yr.

^3^Calculated using reference data for Swedish children [12].

## References

[1] WHO http://www.who.int/dietphysicalactivity/childhood/en/.

[2] Hesketh KD, Campbell KJ (2010). Interventions to prevent obesity in 0–5 year olds: an updated systematic review of the literature. *Obesity*.

[3] Manios Y (2012). The “ToyBox-study” obesity prevention programme in early childhood: an introduction. *Obesity Reviews*.

[4] Fitzgibbon ML, Stolley MR, Schiffer L (2012). Family-based hip-hop to health: outcome results. *Obesity*.

[5] Taveras EM, Gortmaker SL, Hohman KH (2011). Randomized controlled trial to improve primary care to prevent and manage childhood obesity the high five for kids study. *Archives of Pediatrics and Adolescent Medicine*.

[6] Cole TJ, Bellizzi MC, Flegal KM, Dietz WH (2000). Establishing a standard definition for child overweight and obesity worldwide: international survey. *British Medical Journal*.

[7] Larsson I, Forslund HB, Lindroos AK (2004). Body composition in the SOS (Swedish Obese Subjects) reference study. *International Journal of Obesity*.

[8] Ellis KJ, Abrams SA, Wong WW (1999). Monitoring childhood obesity: assessment of the Weight/Height^2^ index. *American Journal of Epidemiology*.

[9] Fields DA, Goran MI, McCrory MA (2002). Body-composition assessment via air-displacement plethysmography in adults and children: a review. *American Journal of Clinical Nutrition*.

[10] Fields DA, Allison DB (2012). Air-displacement plethysmography pediatric option in 2–6 years olds using the four-compartment model as a criterion method. *Obesity*.

[11] Eriksson B, Löf M, Forsum E (2010). Body composition in full-term healthy infants measured with air displacement plethysmography at 1 and 12 weeks of age. *Acta Paediatrica*.

[12] Wikland KA, Luo ZC, Niklasson A, Karlberg J (2002). Swedish population-based longitudinal reference values from birth to 18 years of age for height, weight and head circumference. *Acta Paediatrica*.

[13] McCrory MA, Gomez TD, Bernauer EM, Molé PA (1995). Evaluation of a new air displacement plethysmograph for measuring human body composition. *Medicine and Science in Sports and Exercise*.

[14] Flegal KM, Shepherd JA, Looker AC (2009). Comparisons of percentage body fat, body mass index, waist circumference, and waist-stature ratio in adults. *American Journal of Clinical Nutrition*.

[15] Demerath EW, Fields DA (2012). Challenges in infant body composition. *Pediatric Research*.

